# Inhibitory activities of selected Sudanese medicinal plants on *Porphyromonas gingivalis* and matrix metalloproteinase-9 and isolation of bioactive compounds from *Combretum hartmannianum* (Schweinf) bark

**DOI:** 10.1186/s12906-017-1735-y

**Published:** 2017-04-20

**Authors:** Ebtihal Abdalla M. Mohieldin, Ali Mahmoud Muddathir, Tohru Mitsunaga

**Affiliations:** 1 Faculty of Pharmacy, University of Science and Technology, Omdurman, Sudan; 2 Department of Applied Biological Science, Faculty of Applied Biological Science, Gifu University, 1-1 Yanagido, Gifu, 501-1193 Japan; 3 Department of Horticulture, Faculty of Agriculture, University of Khartoum, Khartoum North-Shambat, Sudan

**Keywords:** Sudanese medicinal plants, *Combretum hartmannianum*, *Porphyromonas gingivalis*, MMP-9, Flavogalonic acid dilacton, Terchebulin

## Abstract

**Background:**

Periodontal diseases are one of the major health problems and among the most important preventable global infectious diseases. *Porphyromonas gingivalis* is an anaerobic Gram-negative bacterium which has been strongly implicated in the etiology of periodontitis. Additionally, matrix metalloproteinases-9 (MMP-9) is an important factor contributing to periodontal tissue destruction by a variety of mechanisms. The purpose of this study was to evaluate the selected Sudanese medicinal plants against *P. gingivalis* bacteria and their inhibitory activities on MMP-9.

**Methods:**

Sixty two methanolic and 50% ethanolic extracts from 24 plants species were tested for antibacterial activity against *P. gingivalis* using microplate dilution assay method to determine the minimum inhibitory concentration (MIC). The inhibitory activity of seven methanol extracts selected from the 62 extracts against MMP-9 was determined by Colorimetric Drug Discovery Kit. In search of bioactive lead compounds, *Combretum hartmannianum* bark which was found to be within the most active plant extracts was subjected to various chromatographic (medium pressure liquid chromatography, column chromatography on a Sephadex LH-20, preparative high performance liquid chromatography) and spectroscopic methods (liquid chromatography-mass spectrometry, Nuclear Magnetic Resonance (NMR)) to isolate and characterize flavogalonic acid dilactone and terchebulin as bioactive compounds.

**Results:**

About 80% of the crude extracts provided a MIC value ≤4 mg/ml against bacteria. The extracts which revealed the highest potency were: methanolic extracts of *Terminalia laxiflora* (wood; MIC = 0.25 mg/ml) followed by *Acacia totrtilis* (bark), *Ambrosia maritima* (aerial part), *Argemone mexicana* (seed), *C. hartmannianum* (bark), *Terminalia brownii* (wood) and 50% ethanolic extract of *T. brownii* (bark) with MIC values of 0.5 mg/ml. *T. laxiflora* (wood) and *C. hartmannianum* (bark) which belong to combretaceae family showed an inhibitory activity over 50% at the concentration of 10 μg/ml against MMP-9. Additionally, MMP-9 was significantly inhibited by terchebulin with IC_50_ value of 6.7 μM.

**Conclusions:**

To the best of our knowledge, flavogalonic acid dilactone and terchebulin were isolated from *C. hartmannianium* bark for the first time in this study. Because of terchebulin and some crude extracts acting on *P. gingivalis* bacteria and MMP-9 enzyme that would make them promising natural preference for preventing and treating periodontal diseases.

**Electronic supplementary material:**

The online version of this article (doi:10.1186/s12906-017-1735-y) contains supplementary material, which is available to authorized users.

## Background

Periodontal diseases are multifactorial infections caused by a specific group of Gram-negative anaerobic bacteria leading to destruction of the tooth-supporting tissue including the alveolar bone and the periodontal ligament. Two major factors contributed to the pathogenesis of periodontitis are namely periodontopathogens which cause direct damage to periodontal tissue through the secretion of toxic products, and the host response to periodontopathogens which results in the release of inflammatory mediators (proinflammatory cytokines, matrix metalloproteinases (MMPs) and prostanoids) [1].

*Porphyromonas gingivalis*, a Gram-negative, black pigmented and an anaerobic bacterium, has been strongly implicated in the etiology of some types of periodontitis including chronic adult periodontitis [2, 3]. It is a major periodontal pathogen that possesses multiple virulence factors including gingipains, lipopolysaccharides and can trigger host cells to release inflammatory cytokines and MMPs [4]. Previous studies showed that MMP-9 secretions were unregulated by *P. gingivalis* supernatant in periodontal ligament fibroblasts, pulp fibroblasts and osteosarcoma cells [5–7].

Most of the Sudanese people in rural areas rely on traditional medicine for the treatment of many infectious diseases (Table 1). Different plant species of medicinal importance have successfully been included in mouthwashes and toothpastes in many countries [8–10]. Human pathogenic microorganisms have developed resistance to drugs owing to the extensive often use of commercial synthetic antibacterial drugs in large quantities without proper medical prescriptions and tests. This condition has raised alarm in most countries and scientists are forced to search for an alternative to these compounds, often in the form of natural medicines from sources such as plants [11].

**Table 1:** Selected Sudanese medicinal plant species used in traditional medicine

No	Botanical names	Family	Vernacular name	Part used	Traditional uses
1	*Calotropis procera* (Aiton) Dryand	Apocynaceae	Ushar	Leaves	Fever, joint pains, muscular spasm, constipation, against scorpion bites, jaundice [45], healing thorn injuries [46], anti-rheumatic [47, 48].
2	*Arestolochia bracteolate* Lam.	Aristochiaceae	Um- Galagel	Whole plant	Malaria, HIV-1 [49, 50].
3	*Xanthium brasilicum* Vell.	Asteraceae	Ramtouk	Leaves	Venereal diseases, malaria [51, 52]
4	*Vernonia amygdalina* Delile		Gharib elwadi	Leaves	Fever, gastro-intestinal disease “GID” [53].
5	*Adanosonia digitata* L.	Bombacaceae	Tabaldi	Fruit pulp	The fruits are used as a cold beverage, added to yoghurt for treatment of diarrhea and amoebic dysentery [54].
6	*Terminalia laxiflora* Engl.	Combretaceae	Darut	Wood	Malaria, cough treatments, heartwood for fumigant [55, 56].
7	*Terminalia brownii* Fresen		Sobagh, Shaff	Wood, bark	Against cough and bronchitis [47], anti-rheumatic [48].
8	*Combretum hartmannianum* (Schweinf)		Habil	Wood, bark	Febrile, jaundice, bacterial infections [37, 38].
9	*Ambrosia maritima* L*.*	Compositae	Damsisa	Aerial part	The herbs are used in treatment of urinary tract infections and elimination of kidney stones, whereas the leaves are used as anti-diabetic and anti-hypertensive [57].
10	*Euphorbia hirta* L.	Euphorbiaceae	Um libina	Aerial part	Decoction of plant is use in asthma and bronchitis [58].
11	*Ricinus communis* L.		Khirwe	Leaves	The leaves are used as a poultice in treatment of abscesses [59, 60].
12	*Acacia seyal* var. fistula (Schweinf.)	Leguminosae	Sfar abide	Wood, bark	Fumigation, rheumatic pain [61].
13	*Acacia seyal* var. seyal Del.		Talih	Wood, bark	Anti-rheumatic, mouth detergent [62].
14	*Acacia tortilis* (Forssk.) Hayne		Seyyal	Wood, bark	Treat skin infection, allergic dermatomes [63].
15	*Cassia acutifolia* Delile		Senna makka	Leaves	Laxative [64], against GID [48].
16	*Parkinsonia aculeata* L.		Sesaban	Leaves	Antipyretic, anti- diabetics [63].
17	*Senna italica* Mill.		Sin elkalb	Leaves	Intestinal complications, haemomorphoids, circulatory system problems, calculi in the urinary system, sexually transmitted diseases [65].
18	*Khaya senegalensis* (Desv) A. Juss	Meliaceae	Mahogany	Bark	Anti-malarial, against hepatic inflammation, sinusitis, skin diseases, GID, trachoma [48].
19	*Polygonum glabrum* Willd	Polygonaceae	Altomsahia	Leaves	Anthelminthic, antimalarial [66].
20	*Argemone mexicana* L.	Papaveraceae	Argemone	Leaves, seed	Venereal diseases [52].
21	*Solanum dubium* Fresen	Solanaceae	Gibben	Fruits	The whole plant and fruits are pulped and applied to wounds and skin tumors as a dressing [67].
22	*Salvadora persica* L.	Salvadoraceae	Alarak	Leaves, stem	Gingivitis, malaria liver swellings, HIV-1 [50, 68, 69].
23	*Tamarix nilotica* (Ehrenb.)Bunge	Tamaricaceae	Tarft al nil	Stem	Febrile, colds [69].
24	*Tribulus terrestri* L.	Zygophyllaceae	Derresia	Aerial part	Demulcent, renal nephritis [47].

*Combretum hartmannianum* a shrub up to 4 m; as a tree under favorable conditions 10 m high. The plant is widespread throughout the Sahel belt from Senegal to Cameroon, and eastwards to the Sudan [12]. Leaves, fruits and stem bark extracts of *C. hartmannianum* showed activity against Gram-positive bacteria, *E. coli* (Gram-negative); and have also been reported to exhibit anti-inflammatory activity [13, 14].

Hence, the purpose of this study was firstly to investigate the antibacterial activity against *P. gingivalis* bacteria of 62 methanolic and 50% ethanolic extracts from 24 selected Sudanese medicinal plants species. Secondly, from these 62 extracts; seven methanol extracts (*Terminalia laxiflora*, *Tamarix nilotica*, *Khaya senegalensis*, *Acacia seyal* var. fistula, *Acacia seyal* var. seyal, *C. hartmannianum* and *Terminalia brownii*) were selected to examine their inhibitory activities against MMP-9 enzyme. Additionally methanolic extract of *C. hartmannianum* bark that demonstrated good combined activities were subjected for further fractionation in order to identify the active compounds responsible for the biological activities.

## Methods

### Plant materials

Twenty four different plant species were collected from Khartoum and Elgadarif States, Sudan, identified and authenticated by Dr. Ashraf Mohamed from the Faculty of Forestry, Mrs. Hamza Tag EL-Sir Herbarium Curator. Voucher specimens (Table 3) were deposited in the Horticultural Laboratory, Department of Horticulture, Faculty of Agriculture, University of Khartoum.

### Preparation of plant extracts

Different plant parts (Table 3) were dried under shade and then grounded before they were subjected to cold maceration with methanol or 50% ethanol. The plant powder was macerated with a gentle shaking for 12 h three times in solvents in side stoppered flasks at room temperature. The extracted solvents were filtrated and evaporated under reduced pressure using a rotatory evaporator, and the concentrated 50% ethanol extracts were then dried with a freeze dryer, resulting in 62 crude extracts and stored at 4 °C until use. In order to prepare stock solution, extracts were dissolved in 100% dimethyl sulfoxide (DMSO). Further serial dilution of the stock was performed to obtain a range of desired concentration of the extracts.

### Fractionation, purification and isolation of *Combretum hartmannianum* bark

Five grams of *C. hartmannianum* bark methanolic extract was subjected to fractionation by medium pressure liquid chromatography (MPLC) using ODS column (YMC-DispoPack AT ODS-25:120 g). The column was conditioned with the first eluent used for separation for 30 min with flow rate 0.5 ml/min. MPLC separation was performed by using a chromatography pump (540 Yamazen, Japan), UV detector at 280 nm wavelength (UV-10 V Yamazen, Japan) and a fraction collector (SF-2120, Advantec Tokyo Ltd., Japan). Elution with H_2_O/MeOH 95/5, 20/80 and absolute methanol resulted in three fractions (F1, F2 and F3). Fraction one (F1), which demonstrated a good inhibitory activity against bacteria and enzyme, was subjected to column chromatography on a Sephadex LH-20 eluted with methanol (90–20%) in water, and finally washed with 70% acetone to give five sub-fractions. Separation of these sub-fractions mainly, (F1–1, F1–2 and F1–3) were performed by using preparative high performance liquid chromatography (HPLC) with reversed phase Inertsil ODS-3 column (GL Sciences Inc. 10 mm i.d. × 250 mm) monitored at 280 nm. The solvent system used was as follows: a gradient program for 60 min from 10 to 100% methanol in water with 0.05% TFA at a flow rate 5 ml/min [15].

Compounds were identified by liquid chromatography-mass spectrometry (LC-MS) with negative ion mode and ^1^H,^13^C NMR. Methanol-d4 was used as the NMR solvent. NMR measurements were obtained by using JEOL ECP 600 MHz NMR. Spectroscopic data of flavogalonic acid dilactone and terchebulin were in good correlation to published data [16, 17] (Table 2).

**Table 2:** ^1^H- and ^13^C–NMR data of flavogallonic acid dilactone and terchebulin (in CD_3_OD) as compared with literature [16, 17]

Position	Flavogallonic acid dilactone					Terchebulin			
	^1^H (ppm) JH,H (Hz)	^1^H (ppm) [16]	^13^C (ppm)	^13^C (ppm) [16]		^1^H (ppm) JH,H (Hz)	^1^H (ppm) [17]	^13^C (ppm)	^13^C (ppm)[17]
								113.0	
					A			125.1	
1			108.1	107.3	B			123.5	
					C			123.5	
					D			122.2	
								114.0	
					A	6.56 (s, H)	6.63 (s, H)	106.8	
2			135.7	135.2	B			113.0	
					C	6.79 (s, H)	6.80 (s, H)	108.5	
					D			141.7	
								138.4	
					A			144.5	
3			136.3	136.2	B			143.4	
					C			144.6	
					D			139.1	
								150.3	
					A			136.1	
4			136.5	136.7	B			135.9	
					C			137.5	
					D			137.6	
								113.0	
					A			143.4	
5	7.26 (s)	7.11(s)	112.8	111.3	B	7.48 (s, H)	7.58 (s, H)	144.5	
					D			144.6	
					C			143.6	
								114.0	
					A			112.0	
6			110.1	109.5	B	6.37 (s, H)	6.37 (s, H)	106.4	
					C			116.0	
					D	6.42 (s, H)	6.39 (s, H)	106.5	
								159.5	160.3
7					A			168.9	169.9
			158.9	157.1	B			169.5	169.1
					C			167.0	167.6
					D			166.9	166.8
1’			108.1	107.3				112.0	
2’			137.8	137.8				114.0	
3’			139.2	138.9				140.7	
4’			143.2	143.1				147.4	
5’			117.5	117.6				113.0	
6’			114.4	109.9				114.0	
7’			160.4	158.9				158.3	157.9
1”			124.9	125.8		5.23 (d, *J* = 2.8 Hz)	5.32 (d, *J* = 4 Hz)	90.2	90.8
2”			120.2	120.0		4.98 (dd, *J* = 3.5, 9.7 Hz)	4.88 (dd, *J* = 3.9 Hz)	74.2	75.1
3”			144.1	143.1		5.64 (t, *J* = 9.6 Hz)	5.59 (t, *J* = 8 Hz)	74.1	74.6
4”			145.9	145.8		4.78 (t, *J* = 11.0 Hz)		68.5	70.1
5”			147.8	147.5		4.21 (t, *J* = 10.3 Hz)		69.0	69.6
6”	7.50 (s)	7.49(s)	113.3	112.5		3.04 (t, *J* = 11.6 Hz) 4.48 (t, *J* = 8.9 Hz)	3.10 (d, *J* = 12 Hz)	63.4	64.4
7”			168.9	167.1		-	-	-	-

### Determination of minimum inhibitory concentration (MIC)

MIC was determined by the broth dilution method according to Iwaki et al. [18]. *Prophyromonas gingivalis* ATTC 33277 was cultured in a Brain-Heart Infusion broth supplemented with 0.5 μg/ml vitamin K and 5 μg/ml hemin. The crude extracts and pure compounds were tested for antibacterial activity in sterile 96-well plates. The inoculums were prepared by diluting the broth culture to approximately 10^8^ cell/ml. To each well; 100 μl of microbial inoculums were added and followed by addition of media to achieve a final volume of 200 μl. The tested extracts or isolated compounds were prepared in a concentration range of 4000–31.3 μg/ml using a two-fold dilution method. The experiments were performed in triplicate. Chlorhexidine was included in the assays as positive control. The cultures were incubated for 72 h at 37 °C under anaerobic conditions. Microbial growth was indicated after the addition of 50 μl of (0.2 mg/ml) p-iodonitrotetrazolium violet (INT) to the cultures and incubated at 37 °C for 2 h. The MIC was defined as the lowest concentration that inhibited the color change of INT [19].

### Measurement of collagenase activity

Collagenase (MMP-9) inhibition activities of seven selected methanolic extracts and isolated compounds were investigated by using a MMP-9 Colorimetric Drug Discovery Kit: AK-404, AK-414 and AK-412 (Enzo Life Science, Plymouth, PA, USA). Briefly, aliquots (50 μl) of buffer solution were distributed into a 96 well plate. Twenty microliter of each diluted MMP-9, methanolic extracts or isolated compounds at different concentrations were added and reaction mixtures were incubated for 30 min at 37 °C and diluted substrate (thiopeptide; 10 μl) was added. N-Isobutyl-N-(4-methoxyphenylsulfonyl) glycyl hydroxamic acid (NNGH) was used as positive control. Inhibition was measured by continuously reading plates at absorbance 414 nm for 10 min in a microplate reader. All assays were performed independently in triplicate [20]. The inhibition of MMP-9 was calculated using the formula:

$$ \mathrm{Inhibition}\%=\left[100\hbox{-} \left(\mathrm{VI}/\mathrm{VC}\right)\right]\times 100 $$

Where:

VI: reaction velocity of (sample or inhibitor).

VC: reaction velocity of control.

### Statistical analysis

The percentage and IC_50_ values of MMP-9 inhibitory activities were expressed as the mean value. The significant differences between extracts or isolated compounds were assessed by one-way analysis of variance (ANOVA) followed by pair wise comparison of the means using Tukey’s multiple comparison test. Values were determined to be significant when *p* was less than 0.05 (*p* < 0.05).

## Results and discussion

In this study methanol and 50% ethanol were chosen as solvent for extraction. As shown in the previous studies, nearly all of the identified components from plants active against microorganisms and enzyme may be related to the polyphenolic content of the plant extract, so the initial screenings of plants can be done by using crude aqueous or alcohol extraction [21, 22].

### Evaluation of MIC activity of plant extracts against *P.gingivalis*

In our search for natural products with beneficial properties for oral health, we evaluated the ability of 24 Sudanese medicinal plants species belonging to 15 families in order to reveal the inhibitory activity against *P.gingivalis* bacteria. Botanical name, part used, voucher specimen and MIC activity of methanol and 50% ethanol extracts against *P. gingivalis* were shown in Table 3. Comparatively, methanol extracts displayed better anti-*P. gingivalis* activity than 50% ethanol extracts. Among 62 plant extracts; 50 extracts exhibited MIC activity at the concentration of 4 mg/ml or less; moderate inhibitory activity (MIC = 1 mg/ml) were found in sixteen plant extracts. The most potent extracts were methanol extract of *Terminalia laxiflora* (MIC value 0.25 mg/ml) followed by *Ambrosia maritima, Argemone mexicana* (seed), *Terminalia brownii* (wood)*, C. hartmannianum* (bark), *Acacia totrtilis* (bark) and 50% ethanolic extract of *T. brownii* (bark) with MIC value 0.5 mg/ml (Table 3). According to the reported previous studies, *T. laxiflora* also showed potent antibacterial activity against *Propionibacterium acne* with MIC value 0.13 mg/ml and their activity was due to hydrolizable tannins [23].

**Table 3:** Minimum inhibitory concentration (MIC) activities of selected Sudanese medicinal plants against *P. gingivalis*

Botanicals name	Examined part	Voucher specimen	MIC mg/ml	
			MeOH	50% EtOH
*A. bracteolate* Lam.	Whole plant	SD-SH-04	2	1
*A. digitata* L.	Fruit pulp	SD-OD-27	2	4
*A. maritima* L*.*	Aerial part	SD-SH-03	0.5	2
*A. mexicana* L.	Leaves	SD-KH-39	-	- ^a^
	Seed		0.5	2
*A. tortilis* (Forssk.) Hayne	Bark	SD-KH-07	0.5	4
	Wood		-	4
*A.seyal* var. fistula (Schweinf.)	Bark	SD-GF-06	1	4
	Wood		-	-
*A.seyal* var. seyal Del.	Bark	SD-GF-05	1	2
	Wood		-	-
*C. hartmannianum* (Schweinf)	Bark	SD-KH-04	0.5	1
	Wood		1	-
*C. procera* (Aiton) Dryand	Leaves	SD-SH-11	2	4
*C.acutifolia* Delile	Leaves	SD-SH-24	1	1
*E. hirta* L.	Aerial part	SD-SH-37	2	4
*K. senegalensis* (Desv) A. Juss	Bark	SD-SH-14	1	2
*P. aculeata* L.	Leaves	SD-SH-02	1	4
*P. glabrum* Willd	Leaves	SD-SH-A-03	1	-
*R. communis* L.	Leaves	SD-SH-36	1	4
*S. dubium* Fresen	Fruits	SD-SH-34	-	2
*S. italica* Mill.	Leaves	SD-SH-25	2	2
*S. persica* L.	Stem	SD-SH-09	1	4
	Leaves		-	-
*T. brownii* Fresen	Bark	SD-GF-02	1	0.5
	Wood		0.5	2
*T. laxiflora* Engl.	Wood		0.25	2
*T. nilotica* (Ehrenb.)Bunge	Stem	SD-OD-10	2	4
*T. terrestri* L.	Aerial part	SD-SH-33	1	1
*V. amygdalina* Delile	Leaves	SD-KH-19	1	2
*X. brasilicum* Vell.	Leaves	SD-SH-12	2	2

^a:^has no activity up to 4 mg/ml, MeoH: Methanol, 50% EtOH: 50% Ethanol

chlorohexidine as positive control has MIC value 0.0004 mg/ml

Also noteworthy the combrataceae family; *C. hartmannianum* (bark), *T. brownii* (wood and bark) and *T. laxiflora* demonstrated inhibitory activity against *P. gingivalis* with MIC values 2 mg/ml or less, except 50% ethanol extract of *C. hartmannianum* (wood) had no activity up to 4 mg/ml. This family has a wide range of tannins, flavonoids, terpenoids and stilbenoids [24, 25]. Flavonoids have been reported to be mainly active against Gram-negative bacteria [26]. In this study, methanolic extract of *C. hartmannianum* (bark) exhibited good activity against *P. gingivalis* (MIC 0.5 mg/ml), and this was in agreement with Eldeen and Van [27] who reported that bark of *C. hartmannianum* inhibited the growth of Gram-negative bacteria at a concentration less than/or around 1.56 mg/ml.

The positive control (chlorhexidine) showed a significant inhibitory activity compared to the other extracts. However, chlorhexidine has several side effects such as undesirable tooth discoloration, unpleasant taste and causing dryness and burning sensation in the mouth, leading to patient dissatisfaction [28, 29].

### Inhibitory activities of selected methanolic plants extracts against MMP-9

Several therapeutic strategies, based on targeting different pathways of the pathogenesis of periodontal disease, have been put forward. In this regard, a number of authors proposed that periodontitis progression could be hampered by successfully inhibiting both bacteria and host-derived proteinases involved in connective tissue destruction of the periodontium [30, 31].

From our previous study to explore a natural agent for preventing and treatment of dental cavity, seven Sudanese methanolic extracts namely; *T. laxiflora* (wood), *Tamarix nilotica* (stem) and bark of *Khaya senegalensis‚ Acacia seyal* var. fistula, *Acacia seyal* var. seyal, *C. hartmannianum* and *T. brownii* showed potent inhibitory activity against glucosyltransferase enzyme that promotes the binding of cariogenic bacteria on the teeth (Additional file 1: Table S1). Therefore these seven methanolic extracts were selected for assayed their ability to inhibit the MMP-9 enzyme.

**Fig. 1:**
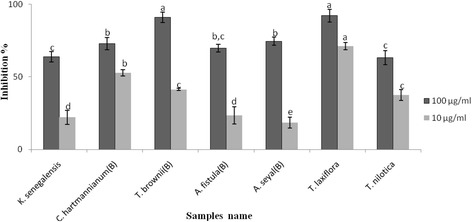
Inhibitory activities of seven methanolic plants extracts against MMP-9. B: bark. NNGH as positive control has 100% inhibition at concentration 100 μg/ml. Values were expressed as mean ± SD, *n* = 3. Values not followed by a common letter were significantly different at the level (*p* < 0.05)

All seven selected extracts exhibited activity higher than 50% inhibition at the concentration of 100 μg/ml against MMP-9 (Fig. 1). NNGH (positive control) recorded 100% inhibition at the concentration of 100 μg/ml. At the concentration of 100 μg/ml, methanolic extracts of *T. laxiflora* and *T. brownii* (bark) significantly inhibited MMP-9. Considerable, but less potent methanolic bark extracts of *A. seyal* var. seyal, *C. hartmannianum* and *A. seyal* var. fistula exhibited MMP-9 inhibitory activity at the concentration of 100 μg/ml. Nevertheless, at the concentration 10 μg/ml, *T. laxiflora* showed the potent inhibitory activity against MMP-9 followed by *C. hartmannianum* (bark). Kusumoto et al. [32] mentioned that the stem bark of *Terminalia arjuna* inhibited the HIV-1 protease activity by more than 70% at a concentration of 0.2 mg/mL. Pomegranate methanol extract inhibited the secretion of MMP-9; this inhibitory effect was likely to be due to hydrolysable tannins [33]. Hydrolyzable tannins were suggested to exhibit their inhibitory effect on the tumor cell invasion via direct inhibition of MMP-9 activity [34]*.* Seigler [35] stated that *Acacia spp*. contained hydrolyzable tannins, flavonoids and condensed tannins.

### Inhibitory activities of compounds isolated from *C. hartmannianium* bark against *P. gingivalis* and MMP-9

In the present study, some plants belong to combretaceae family revealed good inhibitory activities against *P. gingivalis* and MMP-9; such as methanolic extracts of *T. laxiflora*, *C. hartmannianium* (bark) and *T. brownii* (bark). The potency of *T. laxiflora* was probably due to the presence of terchebulin and flavogalonic acid dilactone in wood at high concentration [15]. Kosei et al. [36] isolated gallic acid, punicalagin, terchebulin, ellagic acid 4-O-α-L-rhamnopyranoside, ellagic acid, and 3, 4, 3’-tri-O-methylellagic acid from methanolic extracts of *T. brownii* bark. However, there is no data was reported in literature regarding the isolated compounds from *C. hartmannianium* species bark, which makes it a potential candidate for further separation and isolation of compounds.

Among antimicrobial active compounds isolated from *Combretum spp.* are; combretastatins, acidic tetracyclic and pentacyclic triterpenes/triterpenoids, ellagitannins, phenanthrenes, flavonoids and saponins [37, 38]. *C. hartmannianum* gave good activity against Gram-positive and Gram-negative bacteria, and the most of the activity was found in water and methanol extracts. Additionally, the extracts of *C. hartmannianum* were found to be active against enzymes such as reverse transcriptase and tyrosine kinase [39].

**Fig. 2:**
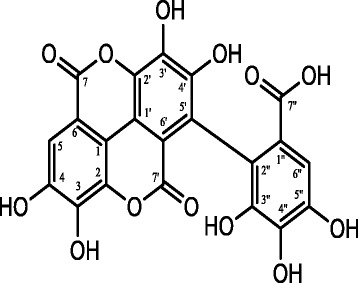
Flavogallonic acid dilactone. Tan powder. LC-MS (negative ion mode) *m/z*: 469 (M-H); ^1^H–NMR (in CD_3_OD): δ (ppm) 7.26 (s), 7.50 (s). ^13^C–NMR (in CD_3_OD): δ (ppm) 108.1 (C-1, 1′), 110.1–114.4 (C-6, 6′), 112.8 (C-5), 113.3 (C-6″), 117.5–120.2 (C-5″), 124.9 (C-1″), 135.7 (C-2), 136.3 (C-3), 136.5 (C-4), 137.8 (C-2′), 139.2 (C-3′), 143.2 (C-4′), 144.1 (C-3″), 145.9 (C-4″), 147.8 (C-5″), 158.9–160.4 (C-7, 7′), 168.9 (C-7″)

**Fig. 3:**
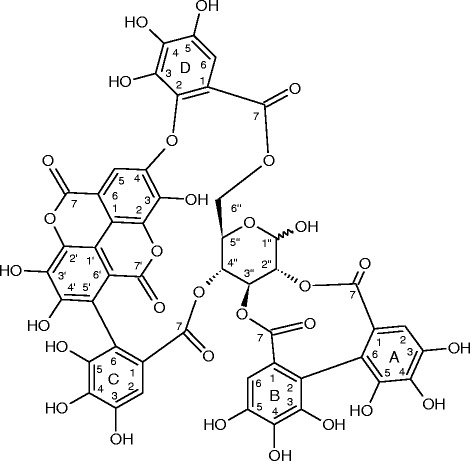
Terchebulin. Tan powder. LC-MS (negative ion mode) *m/z*: 1083 (M-H); ^1^H–NMR (in CD_3_OD): δ (ppm) 3.04 (t, *J* = 11.6 Hz, one of the H-6″), 4.21 (t, *J* = 10.3 Hz, H-5″), 4.48 (t, *J* = 8.9 Hz, one of the H-6″), 4.78 (t, *J* = 11.0 Hz, H-4″), 4.98 (dd, *J* = 3.5, 9.7 Hz, H-2″), 5.23 (d, *J* = 2.8 Hz, H-1″), 5.64 (t, *J* = 9.6 Hz, H-3″), 6.37 (s, H-B6), 6.42 (s, H-D6), 6.56 (s, H-A2), 6.79 (s, H-C2), 7.48 (s, H-5). ^13^C–NMR (in CD_3_OD): δ (ppm) 63.4 (C-6″), 68.5 (C-4″), 69.0 (C-5″), 74.1 (C-3″), 74.2 (C-2″), 90.2 (C-1″), 106.4 (C-B6), 106.5 (C-D6), 106.8 (C-A2), 108.5(C-C2), 112.0–114.0 (C-A6, B2, 5, 5′, 1, 1′, 2, 2′, 6, 6′), 116.0 (C-C6), 122.2 (C-D1), 123.5 (C- B1,C1), 125.1 (C-A1), 135.9 (C-B4), 136.1 (C-A4), 137.5 (C-C4), 137.6 (C-D4), 138.4 (C-3), 139.1 (C-D3), 140.7 (C-3′), 141.7 (C-D2), 143.4–143.6 (C-A5, B3, C5), 144.5–144.6 (C-A3, B5, C3, D5), 147.4 (C-4′), 150.3 (C-4), 158.3 (C-7′), 159.5 (C-7), 166.9 (C-D7), 167.0 (C-C7), 168.9 (C-A7), 169.5 (C-B7)

Bioassay guided fractionation led to the isolation of two compounds namely, flavogalonic acid dilactone and terchebulin (Figs. 2, 3). Terchebulin, and to a lesser extent flavogalonic acid dilactone showed combined activity against *P. gingivalis* and MMP-9 (Table 4). Marquis et al. [40] reported that polyphenols reduced MMP-9 activity and *P. gingivalis* growth; since polyphenols were reported to possess antimicrobial and anti-inflammatory properties, they might be of interest as therapeutic agents for controlling periodontal diseases, which involved both pathogenic bacteria and host immune responses.

**Table 4:** Minimum inhibitory concentration (MIC) and matrix metalloproteinases −9 (MMP-9) inhibitory activities of isolated compounds from *Combretum hartmannianium* bark

Compounds	MIC (μg/ml)	*IC_50_ against MMP-9 (μM)
Terchebulin	500	6.7 ± 1.5^a^
Flavogallonic acid dilacton	1000	36.1 ± 7.5^b^

*IC_50_-Half minimal inhibitory concentration

Means with different letters in the same column were significantly different at the level (*p* < 0.05); *n* = 3

Terchebulin and flavogalonic acid dilactone had moderate antibacterial activity with MIC values of 500 and 1000 μg/ml, respectively. Previous studies showed that flavogalonic acid dilactone, terchebulin and punicalagin isolated from *Terminalia spp.* demonstrated antibacterial activity against *P. acnes* and *Helicobacter pylori* in a range between 125 to 250 μg/ml [15, 41]. Terchebulin demonstrated more potent activity (6.7 μM) than flavogalonic acid dilactone against MMP-9. Moreover, terchebulin has more reliable activity than chlorhexidine that inhibits MMP-9 at the IC_50_ 25.2 μM [42]. Furthermore, Arabaci et al. [43] found that chlorhexidine had a few genotoxic and cytotoxic effects on human lymphocytes. Studies of the in vitro cytotoxic activity on mouse fibroblasts of terchebulin and flavogalonic acid dilactone showed activity at minimum cytotoxic concentration of ≥1500 μg/ml (1348, 3192 μM respectively) [44]. To the best of our knowledge, hydrolysable tannins mainly, terchebulin and flavogalonic acid dilactone were isolated from *C. hartmannianium* bark for the first time during this study.

## Conclusions

Our study demonstrated that some methanolic crude extracts of Sudanese medicinal plants possessed good combined activities against *P. gingivalis* and MMP-9. Moreover, this study provided new information on terchebulin and flavogalonic acid dilactone which were isolated from methanolic extracts of *C. hartmannianium* bark, indicating that they possessed interesting inhibitory properties against *P. gingivalis* and MMP-9, and this may be useful for the prevention and treatment of periodontal diseases. Further studies are recommended to investigate the mechanisms of action of these isolated compounds, toxicity and their usefulness as a source of new components in mouthwashes and toothpastes.

## Additional file

Additional file 1: Table S1. IC50values (μg/ml) of the potent methanolic extracts of Sudanese medicinal plants against glucosyltransferase (GTFs) enzyme. (DOCX 12 kb)
