# Supplementary material for: Inhibitory activities of selected Sudanese medicinal plants on Porphyromonas gingivalis and matrix metalloproteinase-9 and isolation of bioactive compounds from Combretum hartmannianum (Schweinf) bark
Source: BMC Complement Altern Med. 2017 Apr 20;17:224. doi: 10.1186/s12906-017-1735-y (PMC5399347; doi:10.1186/s12906-017-1735-y)
Supplement: Additional file 1: Table S1. — IC50values (μg/ml) of the potent methanolic extracts of Sudanese medicinal plants against glucosyltransferase (GTFs) enzyme. (DOCX 12 kb) [file 12906_2017_1735_MOESM1_ESM.docx]

**Table S1**: IC_50_values (µg/ml) of the potent methanolic extracts of Sudanese medicinal plants against glucosyltransferase (GTFs) enzyme.

| Botanicals name | Part | GTFs  IC_50_(µg/ml) |
| --- | --- | --- |
| *A. seyal var seyal Del* | Bark | 3.8±1.7^a^ |
| *T. brownii* Fresen | Bark | 9.2±3.4^a^ |
| *T. laxiflora* Engl.& Diels | Wood | 13.3±6 ^a,b^ |
| *K. senegalensis* (Desv.) A. Juss | Bark | 23.6±2.6 ^b,c^ |
| *C. hartmannianum* (Schweinf) | Bark | 20.4±4.1 ^b,c^ |
| *A. seyal* var fistula (Schweinf.) | Bark | 47.2±3.6 ^e,f^ |
| *T. nilotica* (Ehrenb.) Bunge | Stems | 49.8±4.9 ^f^ |

Results obtained are expressed as means ±S.D.
